# Association between BDNF G196A (Val66Met) polymorphism and cognitive impairment in patients with Parkinson's disease: a meta-analysis

**DOI:** 10.1590/1414-431X20198443

**Published:** 2019-07-29

**Authors:** Qian Wang, Jianfang Liu, Yikun Guo, Guanzhong Dong, Wenying Zou, Zhuoyou Chen

**Affiliations:** Department of Neurology, the Affiliated Changzhou No. 2 People's Hospital of Nanjing Medical University, Changzhou, Jiangsu, China

**Keywords:** BDNF, Meta-analysis, Cognitive impairment, Parkinson's disease

## Abstract

Brain-derived neurotrophic factor (BDNF) is widely expressed in the central nervous system and prolongs the survival of dopaminergic neurons in the substantia nigra. Several studies have recently investigated the association between BDNF G196A (Val66Met), a single nucleotide polymorphism influencing cognitive processes, and cognitive impairment in Parkinson's disease (PD), but with contradictory findings. Thus, this meta-analysis was performed to clarify the possible association. Relevant studies were identified by a systematic search of PubMed, Embase, and China National Knowledge Infrastructure (CNKI) databases. The strength of the association was evaluated using crude odds ratios and 95% confidence interval. Finally, six studies involving 532 cases and 802 controls were included. Our analyses suggested the G196A (Val66Met) polymorphism was significantly associated with cognitive impairment in PD, especially in Caucasian populations. In conclusion, BDNF G196A (Val66Met) is confirmed to be a risk factor for cognitive impairment in PD.

## Introduction

Parkinson's disease (PD) is a neurodegenerative disease characterized by the degeneration and loss of dopaminergic neurons in the substantia nigra and the formation of Lewy bodies. The main clinical manifestations of PD are tremor, myotonia, bradykinesia, and abnormal posture and gait. Non-motor symptoms (e.g. olfactory dysfunction, cognitive impairment, sleep disorder, pain, depression, anxiety, autonomic nervous dysfunction) are other important clinical manifestations running throughout all stages of PD and occur earlier than motor symptoms, seriously reducing the quality-of-life (1). As one of the most severe and disabling non-motor symptoms of PD, cognitive impairment is closely related to the mortality of the disease and thus has attracted wide attention. Cognitive impairment not only seriously impairs social function and the quality-of-life of PD patients, but also intensifies the burden of caregivers and the costs of disease-related medical care, and extends the length of stay (2). Currently, cognitive impairment in PD patients can be divided into two stages: PD with mild cognitive impairment (PDMCI) and PD with dementia (PDD). Epidemiological data show that the prevalence of PDD is 30%, accounting for 3–4% of the dementia patients (3). About 10% of PD patients develop dementia every year, which is 4–6 times higher than that of non-PD patients (4). PDMCI affects 15–20% of PD patients at early stages; PDD occurs in about 80% of PD patients during the course of the disease (5).

The pathogenesis of PD is still unclear, but is believed to be caused jointly by genetic and environmental factors and to be related to many mechanisms, such as altered protein handling, reactive microgliosis, oxidative stress, and mitochondrial dysfunction (6). The basic pathological changes of PD are the chronic progressive degeneration of dopamine neurons in the substantia nigra and the acidification of the microenvironment around dopamine neurons. PD patients were found with neuronal apoptosis and massive proliferation of glial cells (7).

The exact pathogenesis of cognitive impairment of PD is unclear. Neuropathological findings showed that cognitive impairment of PD was correlated with the number of cortical Lewy bodies, Alzheimer’s disease (AD)-related pathological changes (such as neurofibrillary tangles, senile plaques), and cerebrovascular lesions. At present, the specific susceptibility genes for cognitive impairment of PD are unknown. The brain-derived neurotrophic factor (BDNF), which can significantly enhance the tolerance of dopamine neurons against the acidic environment, is significantly downregulated in the ventral substantia nigra of PD patients (8). G196A (Val66Met, rs6265) polymorphism, with a valine (Val) to methionine (Met) substitution at codon 66, is one of the most frequently studied BDNF gene polymorphisms.

Several recent studies described the association between BDNF G196A (Val66Met, rs6265) polymorphism and cognitive impairment in PD, but reported conflicting and inconclusive findings (9–14). There may be several reasons for the different conclusions. First, diagnostic criteria for cognitive impairment in patients with PD differs greatly. Due to the lack of unified diagnostic criteria, some studies adopt relatively loose criteria, while others adopt more stringent diagnostic criteria, which leads to great differences in research conclusions. Second, dyskinesia such as static tremor, bradykinesia, rigidity, and non-motor symptoms such as pain and fatigue in PD patients may interfere with the accuracy and reliability of cognitive function assessment. Finally, the different age composition and sample size of the subjects will also lead to different research results. In an attempt to resolve these contradictory findings, we performed this meta-analysis involving 6 studies.

## Material and Methods

### Inclusion criteria

Inclusion criteria were: 1) case-control studies investigating the association between gene BDNF G196A (Val66Met) polymorphism and cognitive impairment in PD; 2) sufficient data for calculating the pooled odds ratio (ORs) with 95% confidence interval (CI); 3) studies on humans. Exclusion criteria were 1) duplication; 2) case report or review; 3) article with other genetics polymorphisms and *in vitro* experiments.

### Search strategy

Two investigators independently and systematically searched PubMed, EMBASE, and China National Knowledge Infrastructure (CNKI) databases online up to October 1, 2018 to identify relevant studies using the following keywords: ("BDNF G196A" or "BDNF Val66Met" or "rs6265") AND ("genetic polymorphism" or "single nucleotide polymorphisms" or "SNP") AND ("cognitive impairment" or "dementia") AND ("Parkinson's disease" or "PD"). References in the cited studies and reviews were also checked for other relevant publications. When two studies overlapped, we chose the latest study or the one with a larger sample size. There was no restriction on language, ethnicity, or region of study population. Cognitive impairment was diagnosed according to classification criteria.

### Data extraction and quality assessment

Two investigators reviewed and extracted data independently in accordance with the inclusion criteria. The following information from each study was collected: surname of first author, year of publication, country, ethnicity, sample size, and genotype distributions among cases and controls. When a study involved more than one ethnicity, the genotype data were extracted separately. The quality of the selected studies was assessed using the Newcastle-Ottawa Scale (NOS). Analyses were based on three broad dimensions of selection, comparability, and exposure in the case-control studies. The NOS scores vary from 0 up to 9: high quality (>7); medium quality (4–6); poor quality (<4). Only studies with more than 5 NOS stars were included. The discrepancies were resolved by discussion or consulting with a third reviewer.

### Statistical analysis

Pooled ORs with 95%CI were calculated to evaluate the strength of relationship between BDNF gene rs6265 polymorphism and cognitive impairment in PD. Subgroup analyses were performed according to ethnicity. Heterogeneity between studies was detected by the chi squared-based Q-test. P<0.10 or I^2^>50% was considered to be significant heterogeneity and a random-effects model was applied; otherwise a fixed-effect model was used. Sensitivity analysis was conducted by excluding studies one-by-one to evaluate the effect of individual studies on the pooled ORs and evaluate the stability of the results. A χ^2^ test was used to determine whether the observed genotype frequencies conformed to the Hardy-Weinberg equilibrium (HWE). Publication bias was evaluated by visual inspection of symmetry of Begg's funnel plot and by Egger's test (statistical significance at P<0.05). All statistical analyses were performed on STATA 11.0.

## Results

### Characteristics of the included publications

**Figure 1.**
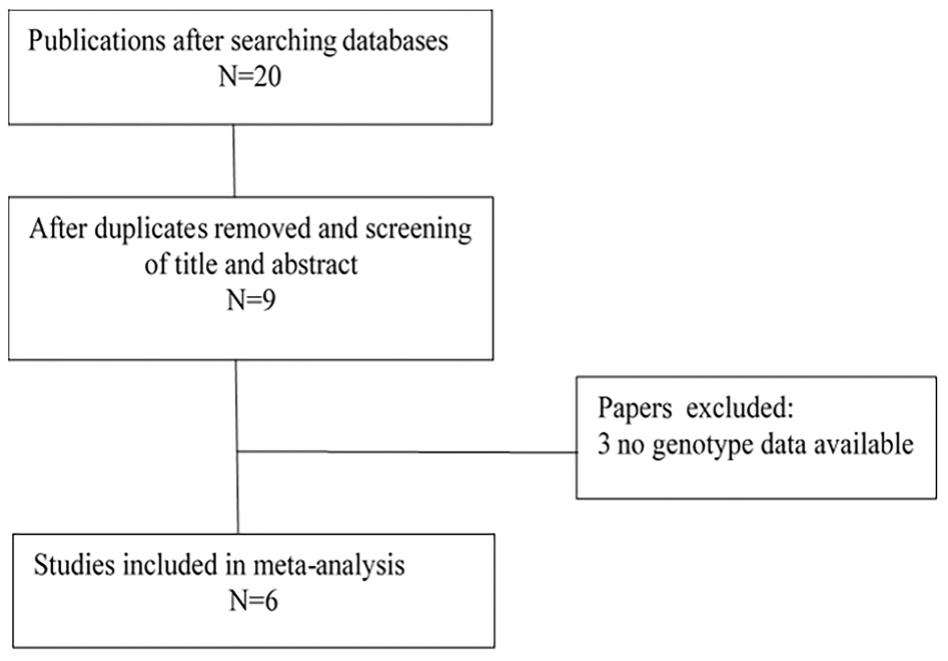
Selection of publications included in this meta-analysis.

The initial search returned a total of 20 articles. Figure 1 represents a flow chart of the retrieved and excluded studies with the reasons for exclusion. Fourteen articles were excluded, including 2 reviews, 9 irrelevant articles, and 3 articles without reporting detailed genotype data. Finally, 6 publications were enrolled, consisting of 532 cases and 802 controls. The characteristics of the selected studies are presented in Table 1. Genotype distributions of rs6265 of the controls conformed to HWE (P>0.05). The NOS scores of the included studies ranged from 5 to 7 stars, suggesting they were of high methodological quality. Five papers were carried out in Caucasian populations and 1 in Asian population.

**Table 1:** Characteristics of included studies.

Author, year (ref n.)	Country	Ethnicity	Case			Control			HWE	NOS
			GG	GA	AA	GG	GA	AA		
Altmann, 2016 (11)	Brazil	Caucasian	56	33	5	61	18	2	0.632	6
Bialecka, 2014 (10)	Poland	Caucasian	143	47	5	33	15	1	0.636	6
Wang, 2014 (12)	China	Asian	54	81	39	22	49	18	0.330	7
Karakasis, 2011 (13)	Greece	Caucasian	10	7	1	105	55	6	0.714	5
Gao, 2010 (14)	Spain	Caucasian	14	6	2	85	63	4	0.052	6
Guerini, 2009 (9)	Italy	Caucasian	12	10	7	145	106	14	0.339	7

HWE: Hardy-Weinberg equilibrium; NOS: Newcastle-Ottawa Scale.

### Quantitative analysis

**Figure 2.**
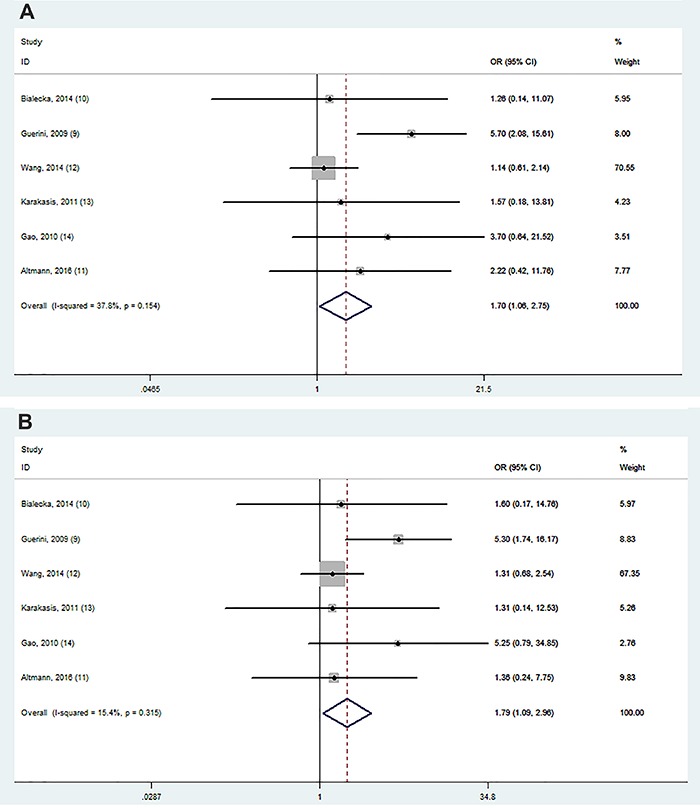
Forest plot showing odds ratio for the associations between rs6265 and cognitive impairment in PD (**A**: AA *vs* GA+GG; **B**: AA *vs* GA).

**Figure 3.**
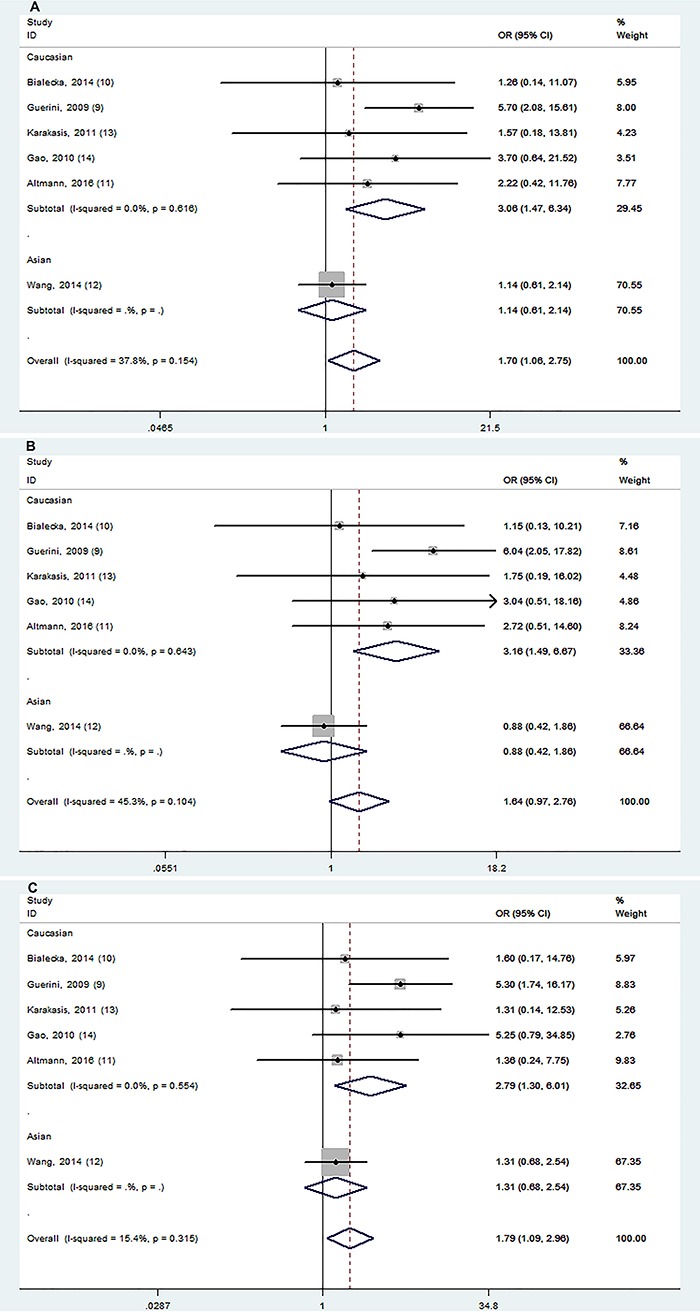
Stratification analyses of ethnicity between rs6265 and cognitive impairment in PD (**A**: AA *vs* GA+GG; **B**: AA *vs* GG; **C**: AA *vs* GA).

Allele and genotype distributions of the BDNF G196A polymorphism from each included study are shown in Table 2. rs6265 polymorphism was significantly associated with cognitive impairment in PD in 2 models (AA *vs* GA+GG and AA *vs* GA; Figure 2). Stratification analyses showed this association was significant among Caucasian populations in 3 models (AA *vs* GA+GG, AA *vs* GG, and AA *vs* GA; Figure 3).

**Table 2:** Meta-analysis of associations between the Val66Met polymorphism and cognitive impairment in patients with Parkinson's disease.

Comparison	Overall and Stratification analyses	Studies	OR (95% CI)	P value	Random/Fixed effect model	P for heterogeneity	I^2^ (%)
A *vs* G	Overall	6	1.32 (0.85,2.07)	0.216	Random	0.002	72.9
	Caucasian	5	1.46 (0.88,2.42)	0.138	Random	0.013	68.5
	Asian	1	0.90 (0.63,1.29)	0.564			
AA *vs* GA+GG	Overall	6	**1.70 (1.06,2.75)**	0.029	Fixed	0.154	37.8
	Caucasian	5	**3.06 (1.47,6.34)**	0.003	Fixed	0.616	0
	Asian	1	1.14 (0.61,2.14)	0.684			
AA+GA *vs* GG	Overall	6	1.09 (0.82,1.46)	0.548	Fixed	0.109	44.4
	Caucasian	5	1.26 (0.90,1.78)	0.179	Fixed	0.166	38.3
	Asian	1	0.73 (0.41,1.30)	0.286			
AA *vs* GG	Overall	6	1.64 (0.97,2.76)	0.062	Fixed	0.104	45.3
	Caucasian	5	**3.16 (1.49,6.67)**	0.003	Fixed	0.643	0
	Asian	1	0.88 (0.42,1.86)	0.743			
AA *vs* GA	Overall	6	**1.79 (1.09,2.96)**	0.022	Fixed	0.315	15.4
	Caucasian	5	**2.79 (1.30,6.01)**	0.009	Fixed	0.554	0
	Asian	1	1.31 (0.68,2.54)	0.423			

Data in bold are significant.

### Sensitivity analysis and publication bias

Sensitivity analysis showed that the omission of any individual study did not substantially influence the risk estimates, which supported the credibility and reliability of this meta-analysis.

Begg's funnel plot or quantitative Egger's test did not find any obvious publication bias for the association between BDNF G196A (Val66Met) and cognitive impairment in PD.

## Discussion

PDMCI is often considered an early manifestation of cognitive decline in PD and a transitional state for the subsequent progression to PDD (15). PDMCI is defined as having at least one cognitive domain (memory, visual space, execution) below 1.5 standard deviations and not meeting the dementia criteria compared with the same age group (16). The pathological study by Braak et al. on PD showed that the lesions of Lewy bodies increased with the prolonged onset time from olfactory bulb and pre-olfactory nucleus to limbic system and neocortex (17). It can be concluded that more brain regions related to cognitive function are damaged as the disease course is lengthened, which, however, cannot explain the cognitive dysfunction in early stage of PD. Moreover, Braak's pathological stage reflects only the deposition of Lewis bodies but not the neuron damage or synaptic dysfunction. AD-related pathological changes (neurofibrillary tangles, senile plaques) play an equally or even more important role than the number of cortical Lewy bodies in the neuropathological changes of cognitive decline of PD (18). In addition, cerebrovascular lesions were confirmed in some PD patients with cognitive dysfunction (19). Imaging and clinical studies showed two possible subtypes of cognitive impairment in PD patients: fronto-striatal deficits (attention, memory, and speech fluency abnormalities featured by relatively slow progression), and posterior-cortical deficits (visual anecdote and memory abnormalities, such as naming and retelling) (20). While fronto-striatal defects are more related to dopaminergic defects and reactive to dopaminergic manipulation, the degeneration of cholinergic projection fibers from the basal forebrain is a highly probable correlate of posterior-cortical defects and is pivotal in PDD development. In addition to dopamine, neurotransmitters such as serotonin, norepinephrine, and choline are also associated with cognitive impairment. Executive function, free recall, and visual spatial ability are associated with abnormal cholinergic function, which may be one of the causes for cognitive impairment in PD.

Because of the family clustering of PDD, it is speculated that there may be some genetic susceptibility to cognitive impairment of PD (21). Although great progress has been made in the study of pathogenic and susceptible genes of PD, the genes related to cognitive impairment of PD have not been fully identified. There are only a few widely recognized susceptibility genes, such as microtubule-associated protein tau (MAPT) gene (22 [Bibr B23]), glucocerebrosidase (GBA) gene23, catechol-O-methyltransferase (COMT) gene (24), dual-specificity tyrosine phosphorylation-regulated kinase 1A (DYRK1A) gene (25), and BDNF gene.

BDNF isolated from pig brain plays an important role in promoting the proliferation, regeneration, synaptic remodeling, and regulated release of neurotransmitters (26). The human BDNF coding gene is located in the short arm p13 of chromosome 11 and consists of 11 exons, which can encode a precursor peptide that is protease-hydrolyzed into mature BDNF protein. The amino acid coding sequence of human BDNF is strikingly similar to that of nerve growth factor (NGF), so BDNF is classified as a member of the NGF family (27). BDNF, which is widely distributed in cortical and subcortical areas, maintains dopaminergic neuronal survival and promotes synaptic plasticity, dendritic morphogenesis, arborization, and even neurogenesis in the adult brain. BDNF is crucial for proper establishment of dopaminergic neurons in the substantia nigra of the developing brain (28). Because BDNF gene participates in many functions of the nervous system, it has been widely studied in cognitive dysfunction and psychobehavioral disorders. G196A (Val66Met) is a frequent single nucleotide polymorphism in the targeting region of the human BDNF and is associated with abnormal intracellular trafficking and the BDNF regulated secretion in the cultured hippocampal neurons transfected with the Met allele. The Met allele is associated with hippocampal neuronal dysfunction and impaired episodic memory in humans (29). The Met allele seems to decrease neuronal dendrites distribution and decrease targeting to secretory granules, lowering the extracellular BDNF levels (30). PD patients that were BDNF Met carriers suffered cognitive impairment more frequently and therefore had lower Mini-Mental State Examination scores than Val/Val homozygotes (9,11). PD patients with BDNF Met alleles, especially women taking dopamine drugs, completed the Tower of London tests better than those with BDNF Val alleles, indicating that BDNF might act on dopamine and its receptors in the limbic system and thereby affect the cognitive function of these patients (31). However, contradictory conclusions were also reported to support a null association (10,12,13,32,33). These discrepancies might be due to different methodologies to assess cognitive impairment. Different cut-offs might have been considered as cognitive impairment in different studies due to differences in years of schooling.

A meta-analysis in 2015 found no association between Val66Met polymorphism and the risk of developing PD in the overall analysis, or between the BDNF G196A genotype and cognitive impairment in the clinical data summarization, but no subgroup analysis of races was performed (34). Lee et al. (35) considered 12 studies in the case of the BDNF 196 G/A polymorphism, and the meta-analysis showed no association between PD and the BDNF 196A allele in all study subjects, however, ethnicity-specific meta-analysis identified an association between the BDNF 196 AA+AG genotype and PD in Europeans, but not in Asians. Zintzaras and Hadjigeorgiou (36) and Dai et al. (37) also found no association between BDNF 196 G/A polymorphism and PD. None of the three studies analyzed the relationship between BDNF and cognitive impairment in PD patients.

We performed this meta-analysis to resolve the contradictory results of all relevant studies published up to 2018. Our analyses suggested that the G196A (Val66Met) polymorphism was significantly associated with cognitive impairment in PD, especially in Caucasian populations. The only study involving the Asian population (12) showed no significant difference in allele or genotype frequencies among non-PD, PDMCI, and PDD groups.

Several potential limitations of this meta-analysis should be considered. First, no subgroup analysis of gender, age of onset, educational level, duration of illness, or genotype methods was conducted owing to the limited number of articles and the small sample size. Second, this meta-analysis only included Asian and Caucasian populations, which calls for future studies on other ethnic groups because of ethnic differences in gene polymorphisms. Third, there was no uniform diagnosis of Parkinson's cognitive impairment in these studies. In 2007, the Movement Disorder Society Task Force (MDS-TF) issued a unified and standardized PDD diagnostic guide and revised it, but the four core cognitive domains were placed in the same weight, and any two cognitive domains with significant impairment could be diagnosed as PDD (38). However, there was also no unified diagnostic criterion for PDMCI. Some studies adopted loose diagnostic criteria, while others used stringent criteria, which led to large differences in the research conclusions. In 2012, MDS-TF issued a new diagnostic guideline for PDMCI, which corresponded to the characteristics of early cognitive impairment of PD and was well consistent with previous PDD diagnostic guidelines (39).

In conclusion, the BDNF gene rs6265 polymorphism plays important roles in cognitive impairment in Parkinson's disease, especially among Caucasian populations. Although cognition cannot be considered a pre-motor biomarker of PD, it acts as a staging and prognostic biomarker and plays a key role in sub-typing and disease stratification. Nevertheless, more large-sample studies following the unified diagnostic criteria for cognitive impairment in PD are needed to further confirm the relationship between BDNF rs6265 and the pathogenesis of PD.
